# Application of Indigenous Rhizospheric Microorganisms and Local Compost as Enhancers of Lettuce Growth, Development, and Salt Stress Tolerance

**DOI:** 10.3390/microorganisms10081625

**Published:** 2022-08-11

**Authors:** Redouane Ouhaddou, Raja Ben-Laouane, Rachid Lahlali, Mohamed Anli, Chayma Ikan, Abderrahim Boutasknit, Aiman Slimani, Khalid Oufdou, Marouane Baslam, Essaid Ait Barka, Abdelilah Meddich

**Affiliations:** 1 Center of Agrobiotechnology and Bioengineering, Research Unit Labelled CNRST (Centre AgroBiotech-URL-7 CNRST-05), Abiotic and Biotic Constraints Team, Cadi Ayyad University, Marrakesh 40000, Morocco; 2 Laboratory of Agro-Food, Biotechnologies and Valorization of Plant Bioresources (AGROBIOVAL), Plant Physiology and Biotechnology Team, Department of Biology, Faculty of Science Semlalia, Cadi Ayyad University (UCA), Marrakesh 40000, Morocco; 3 Phytopathology Unit, Department of Plant Protection, National School of Agriculture of Meknes (ENA), Meknès 50001, Morocco; 4 Laboratory of Microbial Biotechnologies, Agrosciences, and Environment (BioMAgE), Labeled Research Unit CNRST N°4, Faculty of Science Semlalia, Cadi Ayyad University (UCA), Marrakesh 40000, Morocco; 5 Laboratory of Biochemistry, Faculty of Agriculture, Niigata University, Niigata 950-2181, Japan; 6 Resistance and Bio-Protection of Plants Research Unit, University of Reims Champagne-Ardenne, EA 4707-USC INRAE1488, 51100 Reims, France

**Keywords:** arbuscular mycorrhizal fungi, compost, plant growth-promoting rhizobacteria, salinity tolerance, lettuce

## Abstract

This study aimed to mitigate salt stress effects on lettuce by using native biostimulants (arbuscular mycorrhizal fungi (M, consortium), plant growth-promoting rhizobacteria (R, Z2, and Z4 strains), and compost (C)) applied alone or in combination under salinity stress (0, 50, and 100 mM NaCl). Physiological, biochemical, nutritional, mycorrhizal, growth, and soil characteristics were evaluated. Results revealed that growth and physiological traits were negatively affected by salinity. However, mycorrhizal colonization was enhanced under 100 mM NaCl after compost application. The applied biostimulants, particularly M and/or R improved the salinity tolerance of lettuce by increasing the dry biomass by 119% and 113% under 100 mM NaCl, respectively, for M and MR treatments. Similarly, MR enhanced stomatal conductance (47%), water content (260%), total chlorophyll (130%), phosphorus content (363%), and reduced the malondialdehyde (54%) and hydrogen peroxide (78%) compared to the control. Moreover, peroxidase activity (76%) and sugar content (36%) were enhanced by CM treatment, while protein (111%) and proline (104%) contents were significantly boosted by R treatment under 100 mM NaCl. Furthermore, glomalin content was enhanced by MR treatment under severe salinity. In conclusion, the applied biostimulants alone or in combination might help lettuce to tolerate salt stress and enhance its production in degraded areas.

## 1. Introduction

Soil salinization is constantly increasing, affecting 20% of cultivated lands and 33% of irrigated agricultural lands [1], and will affect 50% of land surface by 2050 [2]. Salinity is a major constraint hindering agricultural systems and causes significant soil fertility loss, which limits crop productivity [3]. Three salt stresses are induced in plants, including osmotic, ionic, and oxidative stress. The first causes physiological drought by altering the water potential, thereby reducing water use efficiency by the roots. The second induces an imbalance of mineral elements from the cellular level to the whole plant. The third trigger is reactive oxygen species (ROS) release, which inhibits cell growth and plants’ metabolism [4].

Lettuce (*Lactuca sativa*) is an economically important vegetable crop cultivated worldwide. This crop is characterized by its richness in phytochemicals, including vitamins, carotenoids, antioxidants, and other phytonutrients [5]. However, similar to most crop plants, lettuce is a relatively salt-sensitive crop [6]. Previous studies showed the harmful effects of salinity on lettuce plants in terms of biomass [6,7].

Microorganisms have been studied for their role in reducing salt stress effects. Plant growth-promoting rhizobacteria (PGPR) association to plant roots can induce physiological, growth, and biochemical changes in plants to cope with extreme conditions [8]. PGPR enhances plant growth through biological nitrogen fixation, phosphate solubilization, and the production of siderophores, hydrogen cyanide (HCN), phytohormones, and 1-Aminocyclopropane-1-carboxylate (ACC) deaminase [9]. Rhizobacteria expand plants’ root architecture, thereby facilitating nutrient uptake, improving soil structure, and reducing sodium (Na^+^) absorption [10]. Additionally, they stimulate the biosynthetic pathways of proline and sugars; major osmolytes that help plants withstand the deleterious effects of abiotic stress [11]. The application of arbuscular mycorrhizal fungi (AMF) is also a promising biological approach that can confer resilience to abiotic stress [12]. It has been shown that AMF symbiosis can promote salinity tolerance by improving nutrient uptake [13], photosynthetic activity [14], and water use efficiency [10]. At the metabolic level, AMF symbiosis can protect cell integrity against the deleterious effects of ROS [15].

Similarly, organic supplement represents a promising tool for sustainable and adaptive agriculture [16]. Compost as an organic amendment can improve mineral nutrient uptake, soil organic matter content, soil aggregation, and water holding capacity [17]. Humic substances in compost can chelate sodium through their carboxylic sites [18]. Additionally, combining compost with beneficial microorganisms can improve plant performance under salinity [3].

To the best of our knowledge, there is a lack of data on the effects of the combination of compost, AMF, and PGPR on salinity mitigation in lettuce. Hence, improving lettuce salt tolerance is critical for lowering soil salinity-induced damage. This study focused on utilizing natural AMF, salt-tolerant PGPR strains, and compost as promising approaches to increase lettuce productivity under moderate to high salinity stress. For this purpose, we assessed lettuce growth, physiology, and enzymatic activities under salinity in response to different applications. The impact of these biostimulants and salinity on soil glomalin production was also investigated. By comparing salt-stressed and non-stressed plants, we provide insights into understanding the stress-induced processes in the plant rhizosphere using the microbiome and compost.

## 2. Materials and Methods

### 2.1. Biostimulants Preparation

Two bacterial strains (Z2 and Z4) isolated from the rhizosphere of the Tafilalet palm grove, a semi-arid region located 500 km southeast of Marrakesh, Morocco, were used. To isolate these bacterial strains, collected soil was mixed with a sterile solution of 0.9% NaCl and shacked for 30 min. Serial dilutions were made, and aliquots of dilutions (10^−5^ and 10^−6^) were put on the surface of the National Botanical Research Institute’s phosphate growth medium devoid of yeast extract (NBRIY) medium. The plates were incubated for 48 h at 28 °C. The colonies were further purified by re-streaking on the Tryptone Soy Agar (TSA) medium to obtain single colonies. Each strain was cultivated in Tryptic Soy Broth (TSB) liquid medium under agitation for 48 h at 30 °C until an optical density of approximately 1 at 600 nm (1 × 10^9^ CFU/mL). The molecular characterization of the 16S rDNA gene of the strains showed that Z2 is closely related to *Bacillus* sp., whereas Z4 is identified as *Bacillus subtilis*.

Inoculation was performed by adding 10 mL of the bacterial suspension containing equal volumes of the two abovementioned strains near the roots. After 15 days, a second inoculation was done with the same volume to boost the bacterial level of bacteria in the soil and to ensure infection of newly developed roots. The in vitro plant growth-promoting characteristics of the selected strains were evaluated, and the tolerance of the selected strains to NaCl was examined. To evaluate the effect of salt stress on the multiplication and survival of both bacteria, we prepared solid YEM media at increasing concentrations of NaCl (0, 0.5, 1, and 2 M). Before plating them on Petri dishes divided into equal parts with a loop, we picked and striated the colonies of the bacterial strains onto the agar medium. For each strain, three replicates were performed. After 48 h of incubation at 28 °C, the growth of the strains was evaluated in the Petri dishes. Phosphate and potassium solubilization was performed as reported by Alikhani et al. [19]. Indole-3-acetic acid (IAA) synthesis was tested based on the method developed by Bano and Musarrat [20], while the exopolysaccharide secretion was examined according to the protocol described by Lee et al. [21] (Table 1).

This study used a native AMF consortium isolated from the same region (Tafilalet palm grove). It is constituted of 15 species: *Acaulospora delicata*, *Acaulospora leavis*, *Acaulospora* sp., *Claroideoglomus claroideum*, *Glomus aggregatum*, *G. clarum*, *G. claroides*, *G. deserticola*, *G. heterosporum*, *G. macrocarpum*, *G. microcarpum*, *G. versiforme*, *Glomus* sp, *Rhizophagus intraradices*, and *Pacispora boliviana* [22]. The AMF consortium was propagated for 3 months in pots using *Zea mays* L. as the host plant. The mycorrhizal inoculum consisted of rhizospheric soil from the propagation crop containing mycorrhizal spores, hyphae, and root fragments. Inoculation of lettuce was done by adding 15 g of the inoculum (hyphae, vesicles, roots, and substrate containing spores) to the lettuce root system.

The compost used in the present study is based on olive pomace collected in Marrakesh, Morocco [23]. The maturity of the organic amendment was tested as described by Reddy et al. [24] by calculating the spectroscopic ratio E4/E6 of humic acids of the prepared compost. The physicochemical characteristics of the compost based on the dry matter are mentioned in Table 2.

### 2.2. Plant Growth Conditions

The experiment was performed in a greenhouse, with a day/night cycle of 16/8 h, relative humidity of 68%, an average temperature of 24 °C, and a light of 500 μm^−2^ s^−1^. Lettuce (*Lactuca sativa* L.) seeds were disinfected in 10% (*w/v*) NaClO for 10 min and then cleaned with sterile distilled water. Germination was carried out at 25 °C in Petri dishes containing sterilized filter paper and moistened with sterile distilled water. After 3 days, germinated seeds were transferred into plastic trays filled with sterilized commercial peat in the greenhouse. At the three-leaf age, the seedlings of uniform appearance were transferred into 2 kg plastic bags containing soil pre-sterilized at 180 °C (3 h). The soil used had the following physicochemical properties: sand: 51%, loam: 30%, clay: 19%, available phosphorus: 11 mg kg^−1^, organic matter: 1%, organic carbon: 0.6%, nitrogen: 0.9 mg g^−1^, pH: 8.6, and electrical conductivity: 0.2 mS cm^−1^.

### 2.3. Treatments and Study Design

During the experiment, plants were distributed according to a randomized design and placed randomly in a controlled greenhouse. A factorial design with two main factors; factor 1: salinity conditions testing three levels (0, 50, and 100 mM NaCl), and factor 2: biostimulation treatments at eight levels: (1) Control: plants without any biostimulant, (2) M: plants treated with AMF consortium; R: bacterial consortium treatment; C: compost treatment; MR: AMF consortium and bacterial consortium; RC: bacterial consortium and compost; CM: compost and AMF consortium; and CMR: compost, AMF consortium, and bacterial consortium; was used to establish the experiment. For each treatment, ten replicates were applied. The applied salinity levels were performed based on the germination test of lettuce seeds (Figure S1). Salt stress was applied to lettuce plants 15 days after transplanting. The applied NaCl concentrations were increased stepwise to avoid osmotic shock, starting with 25 mM NaCl for all NaCl-treated pots. The field capacity of all pots was maintained at 75% FC. After 9 weeks of salt treatment (12-week-old plants), mycorrhization analysis, agro-physiological, and biochemical parameters were measured.

### 2.4. Symbiotic Development

After harvesting, root samples from lettuce plants were cut into 1 cm fragments, washed, and cleaned using 10% KOH at 90 °C for 30 min. Then, the samples were treated with 2% HCl for 10 min and stained with Trypan blue at 90 °C for 20 min, as reported by Phillips and Hayman [25]. Mycorrhizal structures’ rate of root infection was done by microscopic observation (ZEISS, Model Axioskop 40 (Carl Zeiss; Oberkochen, Germany)) as described by Trouvelot et al. [26]. The mycorrhization frequency (MF) and intensity (MI) were evaluated by using the following Equations (1) and (2), respectively.

(1) $$\mathrm{Mycorhization}\;\mathrm{Frequency}\;\mathrm{MF}\;\left(\%\right)=\left(\frac{\mathrm{Infected}\;\mathrm{root}\;\mathrm{segment}}{\mathrm{Total}\;\mathrm{root}\;\mathrm{segments}}\right)\times 100$$

(2) $$\mathrm{Mycorhization}\;\mathrm{Intensity}\;\mathrm{MI}\;\left(\%\right)=\frac{\left(95n5+70n4+30n3+n1\right)}{\mathrm{Total}\;\mathrm{root}\;\mathrm{segments}}$$

where (n5, n4, n3, n2, and n1) are the number of fragments denoted 5, 4, 3, 2, and 1, respectively, with the following infection rates: Class 5: more than 91%, Class 4: between 51% and 90%, Class 3: between 11% and 50%, Class 2: less than 10%, Class 1: trace, and Class 0: no mycorrhization.

### 2.5. Growth Measurements and Phosphorus Determination

Leaf number (LN), root length (RL), and shoot height (SH) were determined at the end of harvest. The dry weight was measured after drying the samples at 80 °C until the weight became stable.

The phosphorus (P) content in leaves was estimated by the Olsen method [27].

### 2.6. Photosynthetic Efficiency and Gas Exchanges Measurements

Chlorophyll fluorescence PSII (F_v_/F_m_) was determined using a fluorometer (OPTI-SCIENCE, OS30p, Hudson, NY, USA). Stomatal conductance (g_s_) was measured using a porometer (CI-340, Handheld Photosynthesis System, Washington, DC, USA) as described by Harley et al. [28].

### 2.7. Chloroplastic Pigments Evaluation

Quantification of photosynthetic pigments was determined according to the method described by Arnon [29]. Chlorophyll pigment and carotenoid concentrations were extracted using 80% cold acetone from the fresh tissue powder samples (0.5 g).

### 2.8. Water Content Assessment

The water content (WC) of lettuce plants was determined by the difference between the mass of fresh matter (FM) and that of dry matter (DM) and is expressed by g H_2_O g^−1^ DM using the following formula (3):

(3) $$\mathrm{WC}\;=\frac{\mathrm{FM}-\mathrm{DM}}{\mathrm{DM}}$$

### 2.9. Total Soluble Sugars and Proline Quantification

The concentration of total soluble sugars (TSS) was measured according to the method of Dubois et al. [30]. TSS were extracted by ethanol (80%) (*v/v*) in an aliquot of 0.1 g of the leaf powder previously frozen. After centrifugation, 0.2 mL of supernatant was combined with 0.2 mL of phenol and 1 mL of concentrated sulfuric acid. The amount of TSS was determined by measuring the absorbance at 485 nm.

For proline, fresh samples (0.1 g) were ground in 40% (4 mL) ethanol. The mixture was placed at 4 °C overnight. Then, 0.5 mL of the ethanolic extract was added to 1 mL of a mixture (60% acetic acid, 1% ninhydrin, and 20% ethanol). The formed solution was heated at 90 °C for 20 min. The optical density (OD) was determined at 520 nm [31].

### 2.10. Total Protein Content and Antioxidants Activity Determination

The frozen aerial part (0.1 g) was mixed with 4 mL of 1 M phosphate buffer (pH 7), 2.5% insoluble polyvinylpolypyrrolidone (PVPP), and 0.1 mM EDTA. After centrifugation at 4 °C, the supernatant was used to determine antioxidant enzyme activities [32] and total soluble proteins [33].

Peroxidase (POX) activity was determined according to the method described by Polle et al. [34]. The reaction medium comprised phosphate buffer, 2 mL (pH 7) (0.1 M), 1 mL gaïacol (20 mM), 0.3% H_2_O_2_ (10 mM), and 0.1 mL of the enzyme extract. The reaction started upon adding 0.5 mL of 0.3% H_2_O_2_ (10 mM). POX activity was determined at 470 nm.

Polyphenol oxidase (PPO) activity was measured according to the method of Hori et al. [35]. The reaction medium contained 2 mL of catechol (10 mM) in phosphate buffer (pH 7) and 0.1 mL of enzyme extract. PPO activity was defined as a unit of enzyme mg^−1^ protein. One unit of PPO activity was expressed as the quantity of enzyme, causing an increase in absorbance of 0.001 min^−1^ at 420 nm.

### 2.11. Lipidic Peroxidation and Hydrogen Peroxide Content

Malondialdehyde (MDA) was measured as described by Dhindsa and Matowe [36]. Frozen leaf powder subsamples (0.05 g) were homogenized in 1 mL of 10% trichloroacetic acid (TCA) and 1 mL of acetone (90%). After centrifugation, 0.25 mL of supernatant was added with 0.5 mL of 0.1% phosphoric acid and 0.5 mL of 0.6% thiobarbituric acid (TBA). The solution was incubated at 100 °C for 30 min, and the reaction was stopped by an ice bath. Subsequently, a volume of 0.75 mL of 1-butanol was added. The apparent staining on the layer was measured at 450, 532, and 600 nm.

The concentration of hydrogen peroxide (H_2_O_2_) in the leaves was evaluated following the method described by Velikova et al. [37]. Subsamples of 0.1 g (frozen leaf powder) were mixed with 2 mL of 10% (*w/v*) TCA and centrifuged at 15,000× *g*. The supernatant (0.5 mL) was collected for determination of H_2_O_2_ content, and 0.5 mL of potassium phosphate buffer (10 mM, pH 7) and 1 mL of potassium iodide (1 M) were added. After 3 min of incubation, a standard curve of H_2_O_2_ was elaborated after recording the absorbance at 390 nm.

### 2.12. Soil Quality

At harvest, soil samples were taken from the root zone of the lettuce plants and analyzed. The pH was determined by a pH meter (HI 9025) and the electrical conductivity (EC) using a conductivity meter (HI-9033, Hanna Instruments, Padova, Italy). Available phosphorus (AP) was analyzed according to the method of Olsen and Sommers [27]. Organic matter (OM) and total organic carbon (TOC) were assessed as described by Aubert [38].

Total glomalin-related soil protein (T-GRSP) was examined as described by Cornejo et al. [39]. The extraction of T-GRSP was done from 1 g of soil by adding 4 mL of 50 mM sodium citrate buffer (pH 8.0). The solution was autoclaved for 1 h at 121 °C and centrifugated at 10,000× *g* for 1 h. The T-GRSP content was evaluated according to the Bradford method [33].

### 2.13. Statistical Data Analysis

Results are presented as mean ± SE (standard error) and were treated using analysis of variance (ANOVA) and Tukey’s Honest significant difference test with a significance value of 5%. To determine the interaction between the factors tested (M, R, C, and salinity), a multivariate analysis of variance (MANOVA) was performed using SPSS v. 23 software (IBM, Armonk, NY, USA). Lower numbers indicate significant differences between treatments at the *p* ≤ 0.05 level. All growth, physiological, biochemical, and soil physicochemical characteristics were subjected to a principal component analysis (PCA). The PCA was performed with XLSTAT v. 2014. The heat map was realized with GraphPad^®^ Prism v9.0 (GraphPad Software, San Diego, CA, USA).

## 3. Results

### 3.1. Symbiotic Development

**Figure 1:**
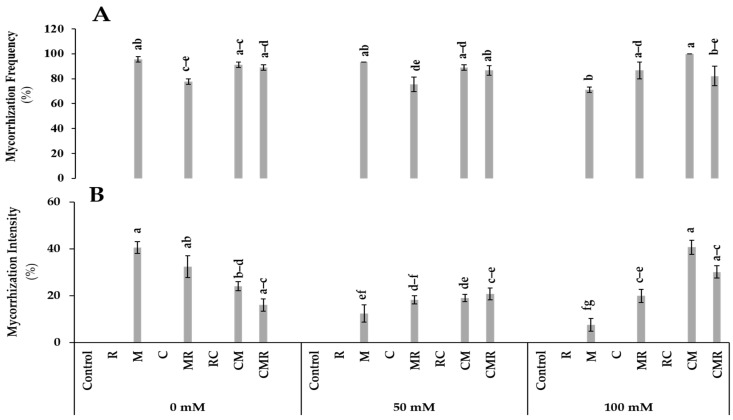
Impact of salt stress (0, 50, and 100 mM NaCl) on (**A**) mycorrhization frequency and (**B**) intensity in lettuce subjected to different biostimulant treatments; control plants (non–amended, non–inoculated), and plants inoculated with plant growth promoting rhizobacteria (R) and/or arbuscular mycorrhizal fungi (M), and/or amended with composts (C). Means (±SE; 5 biological replicates) followed by the same letters are not significantly different at *p* < 0.05 (Tukey’s HSD).

**Figure 2:**
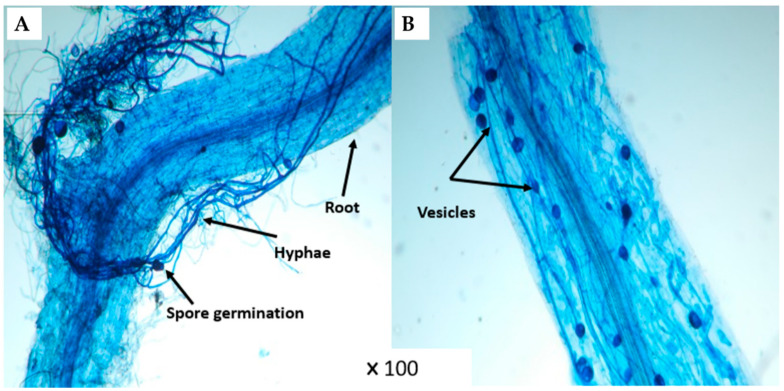
Microscopic observation (×100) of mycorrhizal structures in (**A**) CM and (**B**) CMR treatments of lettuce root.

Non-inoculated plants did not show any colonization on their root systems (Figure 1). No significant difference was observed for mycorrhization frequency under 0 and 50 mM NaCl conditions. This parameter revealed a significant (*p* < 0.001, Table S1) difference in plants treated with compost combined with AMF (CM) (Figure 1A). In addition, CM and CMR treatments decreased the intensity of mycorrhization under non-stressed conditions, while they significantly increased the same parameter under 100 mM NaCl (Figure 1B and Figure 2A,B). The interaction between salinity, M, and C significantly affected both parameters at *p* < 0.001 (Table S1).

### 3.2. Growth Assessment and Mineral Analysis

Salt stress negatively affected all growth parameters, including total dry weight (TDW), shoot height (SH), root length (RL) as well as leaf number (LN) (*p* < 0.001, Table S1). However, the application of biostimulants showed a significant increase in growth parameters compared to the controls under 50 and 100 mM NaCl conditions. LN was significantly increased by M, MR, and CMR treatments compared to control plants under 50 and 100 mM NaCl conditions. R, M, and MR treatments showed a significant difference for SH in the same conditions. RL was significantly enhanced in all plants treated with biostimulants except for R treatment for 100 mM NaCl and CMR for 50 mM NaCl, respectively, compared to the controls (Table 3). Furthermore, the TDW was significantly increased in plants treated by R (141%), M (185%), and MR (241%) under 50 mM NaCl compared to the control. Additionally, under 100 mM NaCl, the TDW recorded high values in plants treated with M, MR, and CMR of 119%, 113%, and 68%, respectively, compared to the control (Table 3). Interaction between salinity, M, and R significantly affected TDW at *p* < 0.05 (Table S1).

**Figure 3:**
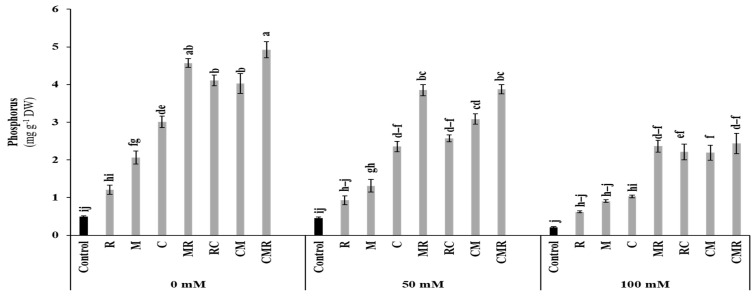
Impact of salt stress (0, 50, and 100 mM NaCl) on P content in lettuce subjected to different biostimulant treatments; control plants (non–amended, non–inoculated), and plants inoculated with plant growth promoting rhizobacteria (R) and/or arbuscular mycorrhizal fungi (M), and/or amended with composts (C). Means (±SE; 5 biological replicates) followed by the same letters are not significantly different at *p* < 0.05 (Tukey’s HSD).

As shown in Figure 3, shoot phosphorus (P) was significantly affected by salinity (*p* < 0.001, Table S1). The obtained results revealed that shoot P concentration was significantly increased in plants treated with MR and CMR under 100 mM NaCl compared to the control. Interaction between salinity and C and salinity and AMF significantly affected shoot P at *p* < 0.001 (Table S1).

### 3.3. Physiological Responses

#### 3.3.1. Photosynthetic Efficiency and Gas Exchanges

**Figure 4:**
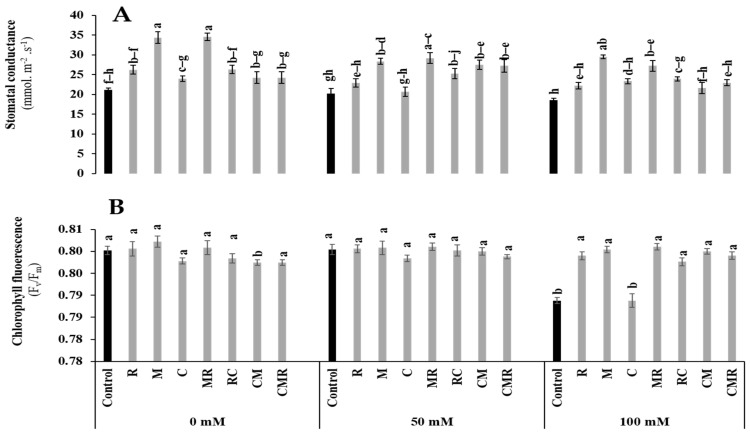
Impact of salt stress (0, 50, and 100 mM NaCl) on (**A**) Stomatal conductance and (**B**) chlorophyll fluorescence in lettuce subjected to different biostimulant treatments; control plants (non–amended, non–inoculated), and plants inoculated with plant growth promoting rhizobacteria (R) and/or arbuscular mycorrhizal fungi (M), and/or amended with composts (C). Means (±SE; 5 biological replicates) followed by the same letters are not significantly different at *p* < 0.05 (Tukey’s HSD).

Stomatal conductance and chlorophyll fluorescence (F_v_/F_m_) were affected by salinity (*p* < 0.05, Table S1). However, the stomatal conductance was significantly increased in plants treated with M, MR, CM, and CMR and M, MR, and RC under 50 and 100 mM NaCl compared to their respective controls (Figure 4A). Under severe conditions, F_v_/F_m_ was significantly enhanced in all treated plants except the C treatment compared to the control (Figure 4B). Interaction between salinity, M, and C had a significant effect on stomatal conductance (*p* < 0.01) (Table S1), while the interaction between salinity, M, and R had a significant effect on F_v_/F_m_ (*p* < 0.05) (Table S1).

#### 3.3.2. Photosynthetic Pigments

**Figure 5:**
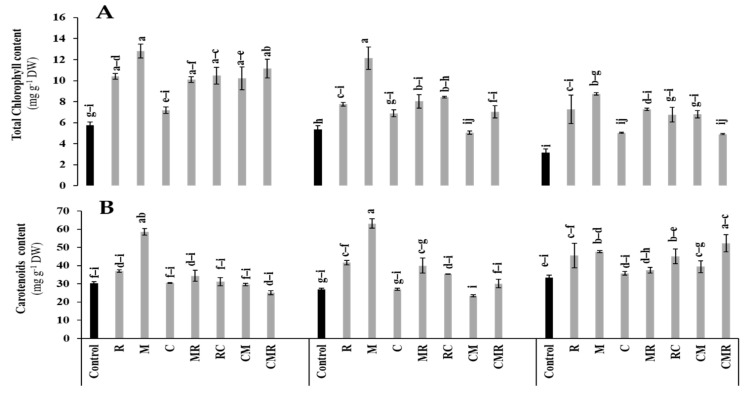
Impact of salt stress (0, 50, and 100 mM NaCl) on (**A**) Total chlorophyll content and (**B**) carotenoids content in lettuce subjected to different biostimulant treatments; control plants (non–amended, non–inoculated), and plants inoculated with plant growth promoting rhizobacteria (R) and/or arbuscular mycorrhizal fungi (M), and/or amended with composts (C). Means (±SE of 5 biological replicates) followed by the same letters are not significantly different at *p* < 0.05 (Tukey’s HSD).

The data presented in Figure 5 showed that the concentrations of total chlorophyll and carotenoid were significantly affected by salinity (*p* < 0.05, Table S1). However, the application of biostimulants alone or in combination positively counteracted the salt stress negative effect compared to the controls (Figure 5). M significantly improved the total chlorophyll concentration under 50 and 100 mM NaCl compared to the control (Figure 5A). Furthermore, the salinity increased the carotenoid content (Figure 5B). Plants treated with R and M revealed a significant increase in carotenoid concentration under 50 mM NaCl. In addition, under 100 mM NaCl, this parameter was significantly improved by R, M, CR, and CMR treatments compared to the control. Interaction between salinity and M significantly affected the content of carotenoids (*p* < 0.01, Table S1).

#### 3.3.3. Water Content

**Figure 6:**
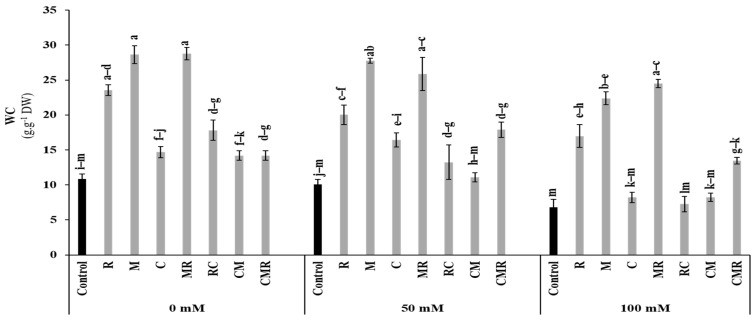
Impact of salt stress (0, 50, and 100 mM NaCl) on water content (WC) in lettuce subjected to different biostimulant treatments; control plants (non–amended, non–inoculated), and plants inoculated with plant growth promoting rhizobacteria (R) and/or arbuscular mycorrhizal fungi (M), and/or amended with composts (C). Means (±SE of 5 biological replicates) followed by the same letters are not significantly different at *p* < 0.05 (Tukey’s HSD).

The water content (WC) of lettuce plants was affected by salt stress (*p* < 0.01, Table S1). However, the application of biostimulants alone and/or in combination significantly improved WC, especially in lettuce plants treated with R, M, MR, and CMR compared to the controls under 50 and 100 mM NaCl conditions (Figure 6). The interaction between salinity, R, and M significantly affected WC (*p* < 0.01, Table S1).

### 3.4. Biochemical Responses

#### 3.4.1. Osmolytes

**Figure 7:**
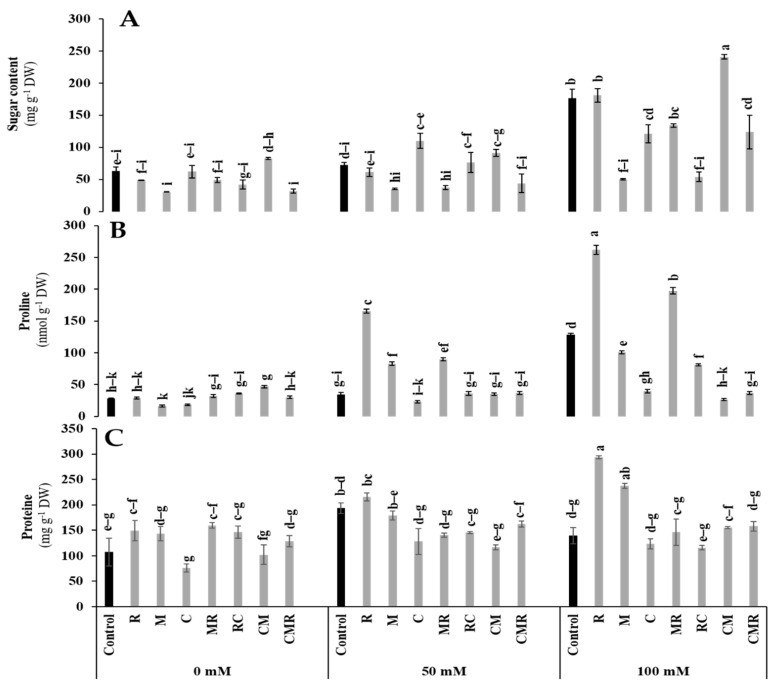
Impact of salt stress (0, 50, and 100 mM NaCl) on (**A**) sugar, (**B**) proline, and (**C**) proteins in lettuce subjected to different biostimulant treatments; control plants (non–amended, non–inoculated), and plants inoculated with plant growth promoting rhizobacteria (R) and/or arbuscular mycorrhizal fungi (M), and/or amended with composts (C). Means (± SE; 5 biological replicates) followed by the same letters are not significantly different at *p* < 0.05 (Tukey’s HSD).

All biochemical parameters were significantly affected by salinity (*p* < 0.01, Table S1). The results revealed that total soluble sugars (TSS) and proline contents were highly accumulated in lettuce plants subjected to salt stress (100 mM NaCl) (Figure 7A,B). The highest value in TSS content was recorded in CM treatment by 36% under 100 mM NaCl (Figure 7A). Indeed, under 50 and 100 mM NaCl conditions, the proline concentration significantly increased in plants inoculated with R (300 and 104%) and MR (158 and 54%), respectively, compared to the controls (Figure 7B). Furthermore, the high salinity exposure of biostimulated and non-biostimulated lettuce plants increased the protein content (Figure 7C). Under the same conditions, plants inoculated with R and M significantly increased the protein content by 111% and 70%, respectively, compared to the control. Interactions between salinity and R and salinity and compost had a significant effect on proline at *p* < 0.01, while the protein content presented a significant effect for salinity, R, M, and C interaction at *p* < 0.01 (Table S1).

#### 3.4.2. Enzymatic Activities

**Figure 8:**
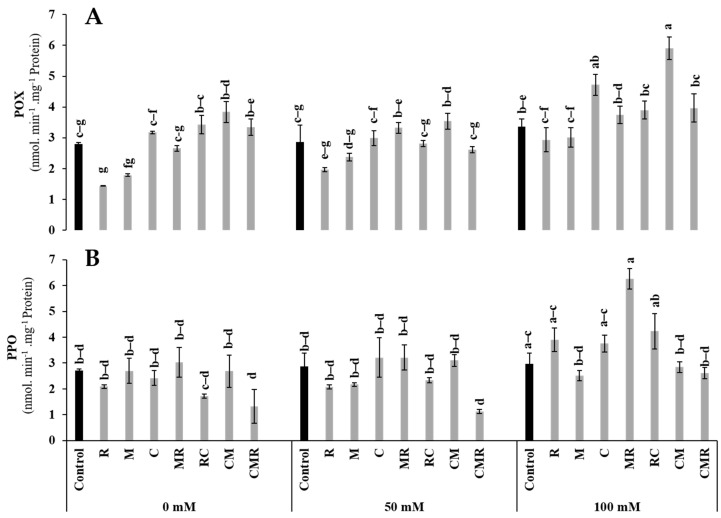
Impact of salt stress (0, 50, and 100 mM NaCl) on (**A**) POX and (**B**) PPO in lettuce subjected to different biostimulant treatments; control plants (non–amended, non–inoculated), and plants inoculated with plant growth promoting rhizobacteria (R) and/or arbuscular mycorrhizal fungi (M), and/or amended with composts (C). Means (±SE; 5 biological replicates) followed by the same letters are not significantly different at *p* < 0.05 (Tukey’s HSD).

The peroxidase (POX) and polyphenol oxidase (PPO) activities were significantly increased under salinity (Figure 8). The POX activity was significantly elevated when lettuce plants were treated with CM under 100 mM NaCl (Figure 8A). Moreover, under the same conditions, PPO activity registered a higher value in MR treatment than in control (Figure 8B).

#### 3.4.3. Stress Markers

**Figure 9:**
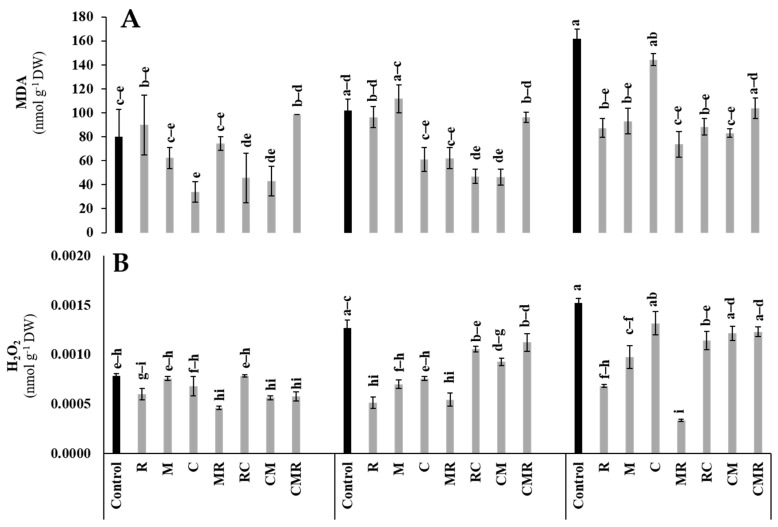
Impact of salt stress (0, 50, and 100 mM NaCl) on (**A**) MDA and (**B**) H_2_O_2_ in lettuce subjected to different biostimulant treatments; control plants (non–amended, non–inoculated), and plants inoculated with plant growth promoting rhizobacteria (R) and/or arbuscular mycorrhizal fungi (M), and/or amended with composts (C). Means (±SE of 5 biological replicates) followed by the same letters are not significantly different at *p* < 0.05 (Tukey’s HSD).

To know the deleterious effects caused by salt stress on lettuce plants, H_2_O_2_ and MDA analyses were performed. An increase in H_2_O_2_ and MDA in the aerial part was remarkable under (100 mM of NaCl) (Figure 9A,B). However, H_2_O_2_ content was significantly decreased in plants treated with R (55%), M (36%), MR (78%), and RC (25%) compared to non-biostimulated plants under 100 mM NaCl (Figure 9A). In the same conditions, MDA content was significantly reduced in plants treated by R (45%), M (42%), MR (54%), RC (45%), and CM (48%) compared to the control (Figure 9B). Interaction between salinity and R significantly affected MDA and H_2_O_2_ at *p* < 0.01 (Table S1).

#### 3.4.4. Soil Quality

Glomalin, available phosphorus (AP), electrical conductivity (EC), pH, total organic matter (TOM), and total organic carbon (TOC) were analyzed after harvesting the lettuce plants (Table 4). Under 100 mM NaCl, these parameters were improved by increasing the glomalin content, especially in the soil treated with MR. A highly significant increase in available phosphorus (AP) was also revealed, especially when C was added or combined with R and/or M. Similarly, stabilization or even a decrease in EC and pH was observed after the application of the biostimulants, mainly when M or R were used alone or combined, or in a tripartite application (CMR) under high salinity (*p* < 0.05, Table S1). An improvement was observed in TOM and TOC, especially when C is added or combined with R and/or M under 100 mM NaCl. Thus, following the application of biostimulants and under salt stress, soil quality was improved compared to the control soils. The interaction between salinity, AMF, and compost significantly affected TOM, TOC, and glomalin at *p* < 0.05 (Table S1).

#### 3.4.5. Principal Component Analysis

**Figure 10:**
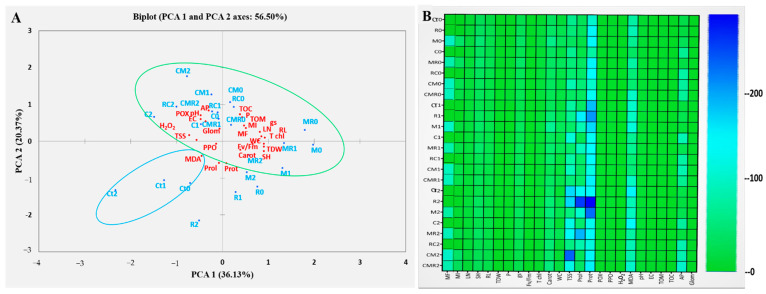
(**A**) Principal component analysis (PCA) and (**B**) Heatmap analyses of lettuce grown without (0 mM NaCl) or with (50 and 100 mM NaCl) salt stress and submitted to different biofertilizer treatments. Ct: control; R: PGPR consortium; M: AMF consortium; C: compost; MR; RC; CM; CMR The numbers 0, 1, and 2 mean 0 mM NaCl, 50 mM NaCl, and 100 mM NaCl, respectively. LN: leaf number; SH: shoot height; RL: roots length; TDW: total dry weight; F_v_/F_m_: chlorophyll fluorescence; gs: stomatal conductance; T Chl: total chlorophyll; WC: water content; Carot: carotenoids; MI: mycorrhizal intensity; MF: mycorrhizal frequency; H_2_O_2_: hydrogen peroxide; MDA: malondialdehyde; Prol: proline; Prot: protein; Sug: sugar; POX: peroxidase activity; PPO: polyphenol oxidase activity; pH: hydrogen potential; EC: electrical conductivity; TOM: organic matter; AP: soil available phosphorus; P: plant phosphorus; Glom: Glomalin; and TOC: total organic carbon.

Principal component analysis (PCA) highlighted how the evaluated parameters were correlated as a function of the applied treatments under normal and saline conditions. PCA results revealed two principal components: PCA1 (36%) and PCA2 (20%) of the total variance (56%) (Figure 10A). Positive correlations were found between the measured parameters and the different biological treatments applied. The traits P, TOC, and TOM, as well as pH, EC, AP, and POX, were positively intercorrelated along the PCA2 axis. In addition, MF, MI, LN, RL, T chl, WC, F_v_/F_m_, TDW, SH, and Carot were positively correlated with each other along the PCA1 axis. The parameters H_2_O_2_, TSS, and MDA, as well as proline and protein, were negatively intercorrelated along the PCA1 and PCA2 axes, respectively.

To detect the fundamental parameters responsible for the positive and negative effects of native biostimulants on lettuce plants under normal and saline conditions, a heat map was used. Figure 10B showed the degree of contribution of each parameter to the tolerance of lettuce under normal and saline conditions; the boxes colored in blue indicate the high parameter value while those colored in green indicate average or low values, respectively. The infection rate of roots and proteins appears to not change with salinity. Under 50 and 100 mM NaCl, most parameters such as proline, proteins, sugars, and soil P appeared contrasted (blue), especially in R, M, MR, and CM treatments under severe and moderate salinity.

## 4. Discussion

**Figure 11:**
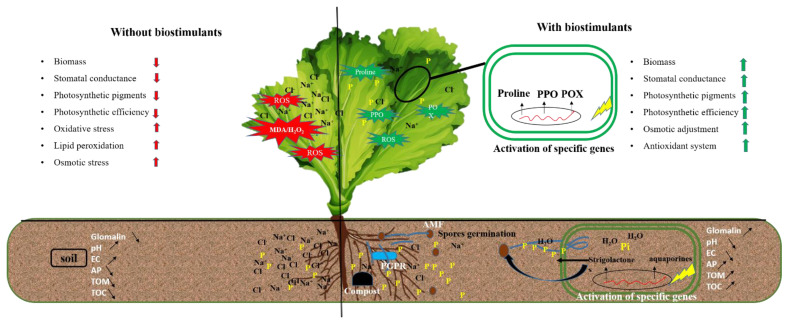
Diagrams summarizing the effects induced by biostimulants (PGPR, AMF, and compost) leading to the tolerance of lettuce to salt stress. POX: peroxidase activity; PPO: polyphenol oxidase; EC: electrical conductivity; AP: soil available phosphorus; TOM: total organic matter; TOC: total organic carbon.

Our results showed that severe salt stress significantly reduced the mycorrhization intensity in roots inoculated with M consortium. As previously mentioned, salinity decreased AMF root colonization through the degradation of mycorrhizal structures [40]. However, Santander et al. [6,10] reported that salinity did not affect AMF root colonization. The success of AMF in colonizing plant roots is regulated by growth hormones such as strigolactone by promoting the branching of fungal hyphae [41,42] (Figure 11). On the other hand, our results showed that compost and/or PGPR increased the mycorrhization intensity under 100 mM NaCl. Compost can stimulate spore germination under salinity via its humic substances [43]. In contrast, Ben-Laouane et al. [44] found that compost decreased mycorrhizal structures under salinity, whereas Cavagnaro [45] reported that plant growth trait improvement is not always proportional to root infection by mycorrhiza. The same scenario was observed in our study, as the growth traits were not often correlated to the degree of AMF root colonization, despite the high colonization observed in plants treated with CM under severe salt stress. The root system infection by M can be related to other factors, including the plant genotypes [46], stress duration [47], and soil structure [48]. Furthermore, our results showed that M and R co-inoculation significantly affected root colonization under salinity. A similar effect was noted by Visen et al. [49] who showed that bacterial consortiums could act as Mycorrhization Helper Bacteria (MHB). The MHB could stimulate plant mycorrhization by increasing the chances and sensitivity of AMF-roots contact. The degree of root colonization by mycorrhizal structures under salinity may be due to the increment of root exudations through the stimulation of the spores’ germination and hyphal elongation [50].

Concerning growth, uninoculated and unamended lettuce plants exposed to 100 mM NaCl hardly survived due to noticeably affected growth and development. However, the application of AMF, compost, and/or PGPR significantly improved all growth parameters. Likewise, better growth of lettuce was observed with RM under salinity. This may be due to the fact that PGPRs accelerate cell division (root) processes by triggering growth hormone biosynthetic pathways (indole-3-acetic acid) [51]. Plant growth improvement induced by AMF could also be related partly to increased P supply, soil water uptake, and soil osmotic potential mediated by AMF [52].

The beneficial effects of AMF, PGPR, and/or compost on lettuce growth could be due to improved mineral nutrient uptake, as evidenced by increased lettuce shoot P content. The improved mineral nutrition is a prominent characteristic of the applied biostimulants [53]. Previous studies showed that the uptake of P by roots is negatively affected by salinity [14] due to its fixation and precipitation with other elements, such as Ca^2+^, Mg^2+^, and Zn^2+^, creating a salt-induced P deficiency in the plants [54]. Our results showed that the P content in lettuce leaves was significantly improved following the application of biostimulants, especially in plants treated with C, MR, RC, CM, and CMR, suggesting a synergistic effect of biostimulants in boosting P uptake by plants in a P-deficiency medium. The P content of lettuce plants treated by MR and CMR was much higher than single treatments under normal and 50 mM NaCl, in which the extra-root hyphae of AMF could absorb the soluble P contained in the compost or delivered by PGPR surrounding the roots (Figure 11). This beneficial impact provided by the M and R treatments in the amended soil with compost could be related to the ability of these microorganisms to acquire slowly released nutrients by the compost [45,55]. Other results could be linked to improving P nutrition with specific P transporters on the surface of fungal hyphae [56]. The P supply may be behind the maximum activity of acid phosphatases secreted by the hyphae of AMF and PGPR [57].

Salinity negatively affects the studied physiological parameters, affecting plant growth. Three successive scenarios might be the leading cause of crop yield reduction: (i) degradation of the thylakoid membrane, followed by (ii) decrease in the photosynthetic machinery, and then (iii) growth decline [58]. In the present study, the reduction in chlorophyll pigments under salinity may be due to the intense activity of chlorophyllases, which alters the photosystem II and, thus, chlorophyll fluorescence [59]. Photosynthetic activity is crucial for biomass productivity but is strongly affected by salinity [6]. The present work revealed that applied biostimulants improved the photosynthetic pigment content, including total chlorophyll and carotenoids. Photosynthetic pigment enhancement is correlated with chloroplasts’ functioning and photosynthetic activity under salinity, especially in the presence of biostimulants [58]. At the thylakoid level, chlorophyll a is considered the central chlorophyll that plays the role of primary electron donor in the reaction center of photosystems. However, it also contributes, together with chlorophyll b and carotenoids, to transferring energy in the antenna complex that will improve the efficiency of photosynthesis [60]. In this study, a carotenoid increase was noted in plants treated with biostimulants in parallel with the severity of salinity in the soil. AMF/PGPR inoculation and/or compost application improve these compounds’ content by stimulating their synthesis pathways [5,61,62].

The increase in NaCl concentration in the soil is accompanied by an increase in ABA and a decrease in the leaf water potential of the chlorophyll content [63]. In agreement with our study, where stomatal conductance was more significant in plants treated with M and MR under 100 mM NaCl conditions, previous studies have highlighted the vital role of AMF in improving the photosynthetic status under abiotic stress [53]. The improved stomatal conductance observed following the application of biostimulants may be related to an increased transpiration rate in the leaves [64]. The opening of stomata is controlled by hormonal regulations such as abscisic acid (ABA) [65]. The symbiotic association of plants with AMF induced upregulation of the expression of chloroplast genes, *RppsbA* and *RppsbD*, under 100 mM NaCl. These genes, in turn, provide the plant with enhanced PSII efficiency and photosynthetic capacity under salinity [66]. The results obtained by [67] also showed that greater chlorophyll pigments represent higher photosynthesis and carbon fixation rates, supporting AMF-plant symbiosis. Indeed, the absorption of water, mineral elements, and the transport of electrons are major attributes guaranteeing a good photosynthetic yield capacity [58]. This could also be attributed to the accumulation of proline and glycine betaine in mycorrhizal plants that protects PSII pigment-protein complexes and CO_2_-fixing enzymes such as RuBisCO and rubisco activase [68]. In the present study, the salt tolerance of lettuce plants may be due to the attenuation of the harmful effects of Na^+^ and Cl^−^ ions by different mechanisms stimulated by microorganisms, such as the sequestration of sodium ions in the vacuole and then their exclusion from the cytosol by specific transporters [45]. Ben-Laouane et al. [44] suggested that the difference in the Na^+^/K^+^ ratio between inoculated and non-inoculated plants highlighted the strategies developed by the alfalfa plant to combat salt stress severity. In the same context of biostimulants, the enrichment of the soil by compost based on green waste has improved attributes related to photosynthesis, such as gas exchange under high salinity [69]. The beneficial effect of compost application on these attributes may be associated with increased N uptake since N is a key component in a variety of photosynthetic enzymes, such as RuBisCO [70]. The beneficial effect of compost application could also be related to the accumulation of glycine betaine and proline in amended seedlings that ensures the stabilization of many enzymes involved in CO_2_ fixation, including RuBisCO and carbonic anhydrase, and protection of pigment-protein PSII complexes [70].

Salinity also affects the water balance of the tissues. Our results showed that lettuce plants were treated with M, R, MR, and CMR by maintaining effective hydraulic conductance under salinity. This improvement could be related to the up-regulation of the root aquaporin genes (AQP) [71]. In addition, water availability enhancement may be linked with plant cell expansion, cell division, stomata opening, and transpiration [14,72]. This abundance of water in the leaves of lettuce may be due to the porosity created by the compost or to the AMF hyphae expansion. The same effect has been observed on plants, notably, lettuce, tomato [12], and the carob tree [47]. This systematic balance is controlled by hormonal signals, including abscisic acid (ABA), jasmonic acid, and strigolactones [73].

The accumulation of osmolytes is the significant response of most plants to abiotic stress [74]. Herein, an accumulation of total soluble sugars (TSS) and proteins were observed under 100 mM NaCl conditions when lettuce plants were amended and inoculated with AMF, making plants more resistant to osmotic stress induced by salt exposure [75]. This higher TSS accumulation induced by AMF was attributed to (i) higher photosynthetic efficiency, (ii) greater activities of α- and β-amylases, sucrose phosphate synthase, and acid invertase; (iii) upper organic acid content; and (iv) higher carbon requirement by AMF [76]. In the present study, proline levels varied according to the salt component, compost application, and AMF and PGPR inoculation. Moreover, under 100 mM NaCl, a pronounced increase in proline was observed in R and MR treatments. The high level of proline observed could be due to the potential of AMF and/or PGPR to stimulate its biosynthesis via upregulation of the activity of the Pyrroline-5-carboxylate synthase (P5CS) gene [77] (Figure 11). Such an increase in proline under salt stress offers beneficial effects to lettuce plants to withstand salinity, in particular, by protecting the photosynthetic apparatus by ensuring the overall cellular integrity (proteins, DNA, and lipids) [78] and/or by keeping a water balance in the plant [22]. The scavenging of reactive oxygen species (ROS) under salinity stress is among the distinctive properties of the present molecule [79]. Many studies have stated the osmotolerant and protective role of proline, which can even be a source of nutrition for the plant [11]. Thus, proline makes lettuce plants healthy, aside from salt damage. Our data indicated that biological treatments responded to soil salt toxicity by increasing protein synthesis in lettuce leaves, especially those treated with M and R separately. This could also be explained by the expression of genes encoding Na^+^ ion transporters (Na^+^ sequestration in the vacuole) [80] and aquaporins for enhanced water uptake [6]. The combination of PGPR and AMF positively affected protein contents under salinity [81].

In the present study, lettuce plants exposed to high NaCl responded significantly by increasing PPO and POX activities through the action of microorganisms, and/or combined with compost, compared to controls. POX activity was higher when the soil was amended with compost combined with AMF, confirming an earlier study [82]. A positive correlation between antioxidant enzymes and salinity was demonstrated by Ben-Laouane et al. [44]. The presence of cellular ROS in large quantities induced by salinity results from an imbalance between their production and their elimination by enzymatic and non-enzymatic detoxification mechanisms, subsequently causing an oxidative explosion, including lipid peroxidation (MDA) and the formation of hydrogen peroxide (H_2_O_2_) (Figure 11). In our survey, the decline in biomass of lettuce under salinity was accompanied by an increase in MDA and H_2_O_2_ levels. However, our data showed that adding biostimulants overcame this oxidative stress on lettuce plants, notably in plants inoculated with M, R, MR, and RC. These lesions could be reduced due to the maximum activities of enzymatic antioxidants and scavenger genes induced by microbes [83].

Salinity significantly affects soil characteristics. Our data reveal that implementing biostimulants alone or in combination in the rhizosphere changes the soil characteristics by stabilizing its pH and electrical conductivity (EC), confirming Ren et al. [84]. Indeed, compost application alone and/or in combination with microorganisms (C, M, CM, MR, RC, and CMR) enriched the soil in total organic matter, total organic carbon, and available phosphorus. Regardless of the source of organic matter provided by the compost, the source of organic carbon in the soil treated by AMF and/or PGPR could be the origin of the high levels of glomalin (GRSP). The data obtained by Baumert et al. [85] have shown that AMF and compost contribute to the stability of soil aggregates, resulting in improved soil physicochemical and biological properties, reflecting unobtrusive nutrition by the roots and therefore a better growth. Glomalin is a glycoprotein produced by AMF and plays an essential role in the improvement of soil structure by retaining soil particles in aggregates and stabilizing them due to its strong sticking power and hydrophobicity, creating porosity in the soil that favors its drainage, aeration that allows good root growth, and a source of respiration for an important microbial activity [86]. In addition, it constitutes a stable form of organic carbon reserves which represent an essential part of the soil’s organic matter [87]. Glomalin is also helpful in sequestering various toxic elements, including Na^+^ ions [88]. The relationship between this glycoprotein and salinity has been reported by Garcia et al. [86]. Our data showed that glomalin levels were proportional to the salt concentration in the soil, especially when the substrate received the double inoculation by MR (Figure 11). This could be due to the bacterial consortium’s intense stimulation of AMF spore germination (R); the protective role of hyphae by glomalin under stress conditions was reported by Atakan and Özkaya [89].

Finally, the beneficial microorganisms colonizing the roots and the organic amendments can affect the soil characteristics, which paradoxically influences plant growth and yield. Figure 10 illustrates possible mechanisms of different natural biostimulants selected to enhance salinity tolerance.

## 5. Conclusions

Rhizosphere enrichment with natural biostimulants has shown their ability to overcome the effect of salinity on lettuce plants through the enhancement of two essential systems: (i) the enzymatic antioxidant system and (ii) the osmotic adjustment system, while paradoxically reducing lipid peroxidation and oxidative stress generated by reactive oxygen species. The engineering of natural biostimulants to improve biochemical, physiological, and growth traits of lettuce under the saline component is effective when applied synergistically. It should be noted that AMF and PGPR co-inoculation was the most potent combination. Therefore, for a vision of sustainability of agricultural systems in the context of climate change, it will be interesting to use these bioinoculants in combination with appropriate organic fertilizers in saline land rehabilitation programs as an appropriate mitigation option.

## Figures and Tables

**Table 1:** Properties of the selected PGPR strains.

Activities	Z2	Z4
Secretion of exopolysaccharides (EPS)	++	++
Solubilization of phosphorus	+	+
Solubilization of potassium	+	+
Auxin synthesis	+	+
Resistance to salinity	++	+

+: low; ++: medium.

**Table 2:** Physico-chemical characteristics of the used compost.

Organic Amendment	pH	EC (mS/cm)	TOC (%)	Maturity (E4/E6)	TOM (%)	AP (ppm)	N (%)	C/N
Olive pomace	9.28	4.20	35.89	2.58	64.61	359.10	2.0851	17.21

EC: electrical conductivity; TOM: total organic matter; TOC: total organic carbon; AP: available phosphorus; C: carbon and N: nitrogen.

**Table 3:** Influence of different applied salinity levels (0, 50, and 100 mM NaCl) on the growth parameters of controls and plants amended with composts (C) and/or inoculated with native arbuscular mycorrhizal fungi (M) and/or bacterial consortium (R).

Treatments	Leaf Number			Shoot Height (cm)			Root Length (cm)			Total Dry Weight (g)		
	0 mM	50 mM	100 mM	0 mM	50 mM	100 mM	0 mM	50 mM	100 mM	0 mM	50 mM	100 mM
Control	9.20 ± 0.37 ^e–g^	9.20 ± 0.37 ^e–g^	7.40 ± 0.40 ^g^	27.0 0 ± 1.75 ^d–i^	21.40 ± 1.79 ^hi^	20.60 ± 0.64 ^i^	15.60 ± 1.37 ^f–h^	14.35 ± 0.59 ^gh^	12.40 ± 1.00 ^h^	0.68 ± 0.04 ^f–i^	0.54 ± 0.02 ^hi^	0.50 ± 0.03 ^i^
R	10.80 ± 0.37 ^a–e^	10.40 ± 0.24 ^a–e^	9.60 ± 0.24 ^d–f^	30.40 ± 1.25 ^c–f^	29.40 ± 1.77 ^c–g^	30.20 ± 1.31 ^c–f^	20.70 ± 0.82 ^b–d^	19.00 ± 1.19 ^c–e^	15.40 ± 0.40 ^f–h^	1.64 ± 0.05 ^a^	1.31 ± 0.03 ^bc^	0.73 ± 0.04 ^f–i^
M	12.20 ± 0.37 ^a^	12.20 ± 0.37 ^a^	10.80 ± 0.37 ^a–e^	43.00 ± 1.25 ^a^	35.40 ± 2.05 ^bc^	33.60 ± 1.08 ^b–d^	21.00 ± 0.85 ^bc^	20.80 ± 0.47 ^b–d^	20.00 ± 0.64 ^b–d^	1.67 ± 0.08 ^a^	1.55 ± 0.04 ^ab^	1.09 ± 0.04 ^c–e^
C	9.81 ± 0.37 ^c–f^	9.40 ± 0.40 ^d–f^	8.20 ± 0.37 ^f–g^	30.80 ± 0.47 ^c–f^	25.40 ± 1.25 ^f–i^	21.60 ± 1.65 ^hi^	17.54 ± 0.43 ^d–g^	18.50 ± 0.42 ^c–f^	17.80 ± 0.64 ^c–f^	0.99 ± 0.06 ^d–f^	0.72 ± 0.09 ^f–i^	0.63 ± 0.03 ^g–i^
MR	11.80 ± 0.20 ^ab^	12.20 ± 0.20 ^a^	11.60 ± 0.24 ^ac^	38.80 ± 1.93 ^ab^	38.40 ± 2.83 ^ab^	33.60 ± 1.08 ^b–d^	25.40 ± 0.62 ^a^	22.40 ± 0.40 ^ab^	19.54 ± 0.25 ^b–d^	1.75 ± 0.07 ^a^	1.85 ± 0.07 ^a^	1.06 ± 0.06 ^c–e^
RC	11.00 ± 0.54 ^a–e^	10.00 ± 0.63 ^b–f^	9.4 ± 0.24 ^d–f^	30.40 ± 0.47 ^c–f^	25.60 ± 0.70 ^e–i^	21.20 ± 1.19 ^hi^	19.40 ± 0.64 ^b–e^	20.60 ± 0.57 ^b–d^	17.80 ± 0.47 ^c–f^	1.23 ± 0.10 ^cd^	0.97 ± 0.08 ^d–f^	0.65 ± 0.03 ^g–i^
CM	10.60 ± 0.40 ^a–e^	10.00 ± 0.31 ^b–f^	10.40 ± 0.24 ^a–e^	29.20 ± 0.81 ^c–g^	23.20 ± 0.70 ^g–i^	25.80 ± 1.73 ^e–i^	17.82 ± 0.39 ^c–f^	17.80 ± 0.64 ^c–f^	17.60 ± 0.64 ^d–g^	1.13 ± 0.07 ^c–e^	0.92 ± 0.08 ^c–e^	0.62 ± 0.04 ^e–h^
CMR	10.60 ± 0.40 ^a–e^	10.60 ± 0.50 ^a–d^	10.40 ± 0.24 ^a–e^	29.20 ± 0.81 ^b–e^	27.40 ± 0.86 ^d–h^	27.40 ± 1.93 ^d–h^	19.80 ± 0.47 ^b–d^	16.10 ± 0.31 ^e–g^	20.70 ± 0.31 ^b–d^	1.13 ± 0.07 ^c–e^	1.12 ± 0.06 ^c–e^	0.84 ± 0.05 ^e–h^

Data are mean ± SE of five biological replicates. Means followed by the same letters are not significantly different at *p* < 0.05 (Tukey’s HSD).

**Table 4:** Soil physicochemical analysis after harvest of lettuce grown under different salinity concentrations (0, 50, and 100 mM NaCl) of the control (unamended and uninoculated) and biofertilizer plants with composts (C), and/or arbuscular mycorrhizal fungi (M), and/or plant growth promoting rhizobacteria (R).

Treatments	Glomalin (mg.kg^−1^ DW)			AP (mg. kg^−1^)			EC (mS/cm)			pH			TOM (%)			TOC (%)		
	0 mM	50 mM	100 mM	0 mM	50 mM	100 mM	0 mM	50 mM	100 mM	0 mM	50 mM	100 mM	0 mM	50 mM	100 mM	0 mM	50 mM	100 mM
Control	0.22 ± 0.05 ^i^	0.77 ± 0.09 ^e–i^	0.71 ± 0.03 ^g–i^	19.17 ± 0.14 ^f–h^	11.69 ± 1.45 ^gh^	18.41 ± 0.76 ^fh^	1.24 ± 0.00 ^g^	1.66 ± 0.05 ^ab^	1.50 ± 0.00 ^b–f^	7.80 ± 0.07 ^g–i^	7.90 ± 0.03 ^c–h^	7.95 ± 0.01 ^b–g^	1.16 ± 0.05 ^f–h^	1.07 ± 0.05 ^gh^	0.88 ± 0.02 ^h^	0.67 ± 0.03 ^f–h^	0.62 ± 0.03 ^gh^	0.51 ± 0.01 ^h^
R	0.27 ± 0.07 ^i^	0.82 ± 0.06 ^e–i^	2.01 ± 0.06 ^b–e^	14.38 ± 2.01 ^f–h^	24.12 ± 6.27 ^fg^	11.61 ± 0.29 ^gh^	1.31 ± 0.02 ^fg^	1.34 ± 0.06 ^fg^	1.26 ± 0.03 ^g^	7.76 ± 0.00 ^hi^	7.75 ± 0.01 ^i^	7.75 ± 0.00 i	1.56 ± 0.02 ^c–f^	1.71 ± 0.02 ^a–e^	1.16 ± 0.04 ^f–h^	0.91 ± 0.01 ^c–f^	0.99 ± 0.01 ^a–e^	0.67 ± 0.02 ^f–h^
M	0.58 ± 0.16 ^hi^	1.24 ± 0.05 ^c–i^	1.80 ± 0.20 ^b–h^	27.06 ± 1.84 ^f^	11.02 ± 0.46 ^h^	23.45 ± 2.69 ^f–h^	1.29 ± 0.01 ^fg^	1.42 ± 0.06 ^d–g^	1.33 ± 0.01 ^fg^	7.80 ± 0.02 ^g–i^	7.81 ± 0.01 ^f–i^	7.82 ± 0.01 ^e–i^	2.03 ± 0.15 ^ab^	1.85 ± 0.04 ^a–d^	1.65 ± 0.00 ^b–e^	1.18 ± 0.08 ^ab^	1.07 ± 0.02 ^a–d^	0.96 ± 0.00 ^b–e^
C	0.75 ± 0.18 ^f–i^	1.97 ± 0.66 ^b–f^	2.75 ± 0.44 ^ab^	64.60 ± 0.80 ^de^	86.93 ± 2.20 ^b^	72.91 ± 1.07 ^c–e^	1.23 ± 0.00 ^g^	1.25 ± 0.00 ^g^	1.35 ± 0.04 ^e–g^	7.97 ± 0.04 ^b–e^	7.92 ± 0.03 ^b–g^	8.00 ± 0.03 ^a–d^	2.03 ± 0.01 ^ab^	1.99 ± 0.03 ^ab^	2.07 ± 0.03 ^a^	1.18 ± 0.00 ^ab^	1.16 ± 0.02 ^ab^	1.20 ± 0.02 ^a^
MR	1.24 ± 0.01^c–i^	2.64 ± 0.33 ^ab^	3.56 ± 0.27 ^a^	15.72 ± 0.36 ^f–h^	23.20 ± 5.79 ^f–h^	14.97 ± 0.85 ^f–h^	1.27 ± 0.03 ^g^	1.25 ± 0.02 ^g^	1.29 ± 0.02 ^fg^	7.85 ± 0.00 ^d–i^	7.82 ± 0.01 ^e–i^	7.82 ± 0.02 ^e–i^	2.04 ± 0.19 ^ab^	1.72 ± 0.00 ^a–e^	1.70 ± 0.00 ^a–e^	1.18 ± 0.11 ^ab^	1.00 ± 0.00 ^a–e^	0.99 ± 0.00 ^a–e^
RC	1.08 ± 0.18 ^d–i^	2.72 ± 0.08 ^ab^	1.90 ± 0.12 ^b–g^	77.44 ± 1.67 ^b–d^	73.83 ± 4.92 ^c–e^	87.86 ± 1.21 ^b^	1.69 ± 0.01 ^ab^	1.58 ± 0.01 ^a–d^	1.77 ± 0.00 ^a^	8.04 ± 0.01 ^a–c^	7.94 ± 0.01 ^b–g^	8.13 ± 0.01 ^a^	1.96 ± 0.08 ^a–c^	2.08 ± 0.07 ^a^	1.88 ± 0.01 ^a–d^	1.13 ± 0.05 ^a–c^	1.21 ± 0.04 ^a^	1.09 ± 0.00 ^a–d^
CM	0.91 ± 0.03 ^d–i^	2.06 ± 0.08 ^b–d^	2.36 ± 0.34 ^bd^	70.31 ± 1.76 ^c–e^	83.15 ± 0.52 ^c–e^	88.70 ± 1.01 ^bc^	1.65 ± 0.02 ^a–c^	1.73 ± 0.02 ^a^	1.73 ± 0.04 ^a^	7.97 ± 0.02 ^b–d^	7.86 ± 0.01 ^d–i^	8.06 ± 0.04 ^ab^	1.81 ± 0.05 ^a–d^	2.09 ± 0.02 ^a^	2.11 ± 0.16 ^a^	1.05 ± 0.02 ^a–d^	1.21 ± 0.01 ^a^	1.22 ± 0.09 ^a^
CMR	0.88 ± 0.11 ^d–i^	1.80 ± 0.22 ^b–h^	1.95 ± 0.18 ^g–i^	67.37 ± 0.51 ^de^	61.91 ± 2.14 ^e^	105.58 ± 0.38 ^a^	1.56 ± 0.08 ^a–e^	1.72 ± 0.08 ^a^	1.44 ± 0.04 ^c–g^	7.95 ± 0.01 ^b–f^	7.95 ± 0.01 ^b–g^	7.86 ± 0.00 ^d–i^	1.35 ± 0.08 ^e–g^	1.53 ± 0.06 ^d–f^	1.97 ± 0.05 ^a–c^	0.78 ± 0.04 ^e–g^	1.14 ± 0.03 ^d–f^	1.14 ± 0.03 ^a–c^

Data are mean ± SE of five biological replicates. Means followed by the same letters are not significantly different at *p* < 0.05 (Tukey’s HSD).

## Data Availability

Data generated in this study are available upon reasonable request to the corresponding author.

## Supplementary Materials

The following supporting information can be downloaded at: https://www.mdpi.com/article/10.3390/microorganisms10081625/s1, Figure S1: Germination rate of lettuce seeds according to different NaCl concentrations; Table S1: Results of multivariate analysis of variance (MANOVA) test for independent variables including salt stress (SS), arbuscular mycorrhizal fungi (AMF), plant growth promoting rhizobacteria (PGPR) and compost and their interactions.
