# Supplementary material for: Application of Indigenous Rhizospheric Microorganisms and Local Compost as Enhancers of Lettuce Growth, Development, and Salt Stress Tolerance
Source: Microorganisms. 2022 Aug 11;10(8):1625. doi: 10.3390/microorganisms10081625 (PMC9416567; doi:10.3390/microorganisms10081625)
Supplement: Supplementary file 1 [file microorganisms-10-01625-s001.zip › microorganisms-1846612-supplementary.pdf]

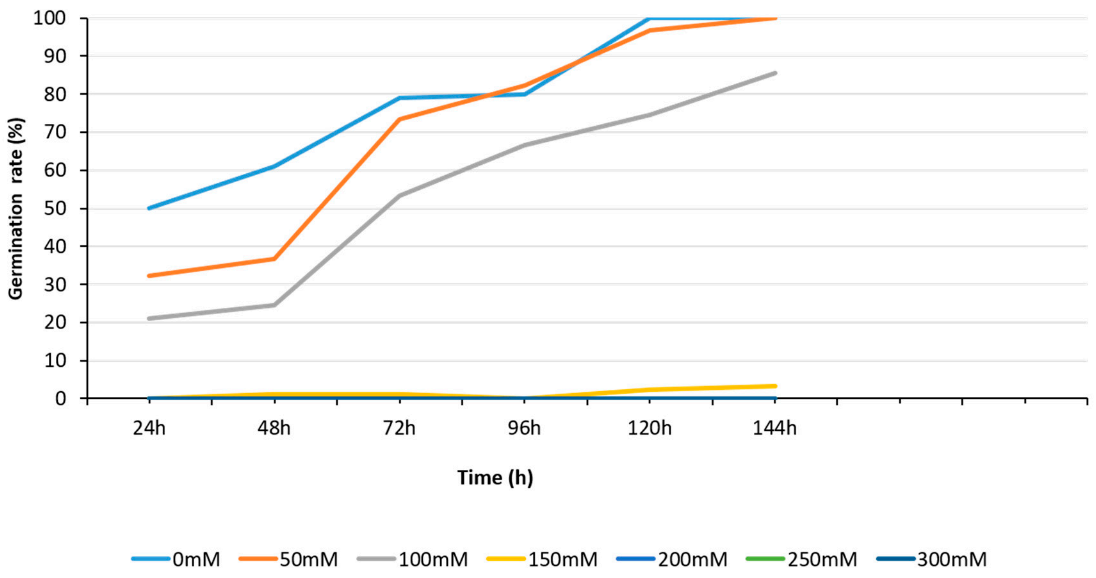

Figure S1. Germination rate of lettuce seeds according to different NaCl concentrations

Table S1. Results of multivariate analysis of variance (MANOVA) test for independent variables including salt stress (SS), arbuscular mycorrhizal fungi (AMF), plant growth promoting rhizobacteria (PGPR) and compost and their interactions.

Treatments	SS	PGPR	AMF	Compost	SS* PGPR	SS* AMF	SS* Compost	PGPR* AMF	PGPR* Compost	AMF* Compost	PGPR*AMF* Compost	SS*PGPR*AMF	SS*AMF* Compost	SS*PGPR* Compost	SS* PGPR*AMF* Compost
MF%	NS	*	***	NS	NS	NS	NS	*	NS	NS	NS	NS	NS	NS	NS
MI%	**	NS	***	NS	*	**	***	NS	NS	NS	NS	*	***	NS	NS
LN	***	***	***	NS	NS	*	NS	*	NS	**	*	NS	NS	NS	NS
SH	***	**	***	***	*	NS	NS	*	NS	***	***	NS	*	*	NS
RL	***	***	***	*	*	NS	**	NS	**	***	NS	NS	NS	NS	NS
TDW	***	***	***	***	NS	NS	NS	***	**	***	**	*	NS	NS	NS
P	***	***	***	***	NS	***	**	**	*	***	***	NS	NS	NS	NS
gs	***	**	***	***	NS	NS	***	**	NS	***	NS	NS	**	NS	NS
Fv/Fm	*	*	**	**	NS	*	NS	*	NS	NS	NS	*	NS	NS	NS
T Chl	***	**	***	NS	NS	*	NS	***	NS	***	**	NS	NS	NS	NS
Carot	*	NS	**	***	*	NS	NS	***	*	***	***	NS	NS	NS	NS
WC	***	***	***	***	NS	NS	NS	**	*	***	***	*	NS	NS	NS
TSS	***	NS	NS	NS	NS	NS	NS	NS	**	*	NS	NS	NS	NS	NS
Prol	***	***	NS	***	**	NS	***	*	**	NS	NS	NS	NS	*	NS
Prot	***	**	NS	***	NS	NS	NS	**	NS	NS	*	NS	*	NS	**
POX	**	NS	NS	***	NS	NS	NS	NS	NS	NS	**	NS	NS	NS	NS
PPO	**	NS	NS	NS	NS	NS	NS	NS	**	*	*	NS	NS	NS	NS
H_2O_2	***	***	**	**	*	NS	**	NS	***	*	NS	*	**	*	NS
MDA	***	NS	NS	**	**	NS	NS	*	*	**	*	NS	NS	NS	NS
pH	NS	NS	NS	***	NS	NS	NS	NS	NS	NS	**	NS	NS	NS	NS
EC	**	NS	*	***	**	NS	NS	***	***	***	***	NS	NS	NS	NS
TOM	NS	NS	*	***	NS	NS	***	***	***	***	NS	NS	**	NS	NS
TOC	NS	NS	*	***	NS	NS	***	***	***	***	NS	NS	**	NS	NS
AP	*	NS	NS	***	NS	NS	**	NS	NS	NS	NS	NS	NS	NS	NS
Glo	***	*	**	*	NS	NS	NS	NS	**	***	*	NS	*	*	NS

MF: Myorrhization frequency; MI: Mycorrhization intensity; LN: leaf number; SH: shoot height; RL: roots length; TDW: total dry weight; P: phosphorus in plant; gs: stomatal conductance; Fv/Fm: chlorophyll fluorescence; T Chl: total chlorophyll; Carot: carotenoids; WC: water content; TSS: Total soluble sugars; Prol: proline; Prot: protein; POX: peroxidase activity; PPO: polyphenol oxidase activity; H_2O_2: hydrogen peroxide; MDA: malondialdehyde; pH: hydrogen potential; EC: electrical conductivity; TOM: total organic matter; TOC: total organic carbon; AP: available phosphorus; Glo: Glomalin; SS: salt stress; NS: not significant, *: $P < 0.05$, **: $P < 0.01$ and ***: $P < 0.001$.
